# A novel cuproptosis-related lncRNA nomogram to improve the prognosis prediction of gastric cancer

**DOI:** 10.3389/fonc.2022.957966

**Published:** 2022-08-29

**Authors:** Anqi Feng, Lingnan He, Tao Chen, Meidong Xu

**Affiliations:** Endoscopy Center, Shanghai East Hospital, Tongji University School of Medicine, Shanghai, China

**Keywords:** lncRNAs, prognostic model, gastric cancer, metabolism, bioinformatics, computational biology, cuproptosis

## Abstract

**Background:**

Cuproptosis is a copper-triggered modality of mitochondrial cell death and cuproptosis process may play important roles in gastric cancer development. However, little is known about cuproptosis-related lncRNAs in gastric adenocarcinoma (STAD). This study is aimed to investigate the potential prognostic signatures of cuproptosis-related lncRNAs in STAD.

**Methods:**

The Cancer Genome Atlas (TCGA) database were used to obtain gene expression profiles, clinicopathological, and OS information for STAD. Cuproptosis-related genes were collected based on previous studies and cuproptosis-related lncRNAs were screened out by co-expression analysis. The nomogram constructed by Cox regression analysis with the minimum absolute contraction and selection operator (lasso) algorithm. In addition, the potential response of ICB therapy and immune evasion incidence were estimated with Tumor Immune Dysfunction and Exclusion (TIDE) algorithm. Immune checkpoint expressions associated with risk scores were also analyzed. The correlation of immune checkpoint CD209 and HAVCR2 expressions associated with risk scores were experimentally testified by RT-qPCR, Western Blot, and IHC.

**Results:**

Patients were classified into high-risk and low-risk groups based on the risk score calculated in this model. The Kaplan–Meier survival curve analysis revealed that the high-risk group was associated with poor prognosis. Multivariate Cox regression analysis suggested that this lncRNA prediction model was an independent risk factor affecting the OS rate. Furthermore, ROC curve indicates that the nomogram was superior to traditional clinicopathological features in predicting STAD prognosis. Finally, functional enrichment analysis and immune checkpoint investigation revealed that the nomogram is notably associated with cholesterol metabolism and immune functions, RT-qPCR and Western Blotting demonstrated the co-expression relationship of LINC01150 with CD209 and HAVCR2.

**Conclusion:**

A novel cuproptosis-related lncRNAs signature impacts on the prognosis and immunological features of GC.

## Introduction

Gastric cancer (GC) is one of the most common malignant tumors worldwide, and gastric adenocarcinoma (STAD) is the predominant histological type of gastric cancer (1). Global Cancer Statistics 2020 reports that there were estimated 769,000 GC deaths, and GC ranked fifth in incidence and forth in mortality among malignant tumors (2). Since gastric cancer is often diagnosed at an advanced stage, it remains poorly prognosed (3, 4). The existing criteria for evaluating the prognosis of patients can no longer meet the needs of clinical diagnosis and treatment. Thus, it is particularly critical to explore new prognostic prediction methods.

Over 80% of the human genome is transcribed as noncoding RNAs with biological functions, including long noncoding RNAs (lncRNAs) (5). LncRNA, as an RNA transcript of more than 200 nucleotides, can act as an oncogene or tumor suppressor gene to play a complex regulatory role in the occurrence and development of tumors (6). LncRNAs are also of great significance in predicting tumor prognosis. Subtle regulation of lncRNA expression can provide signals of transformations in tumor malignancies (7). One study constructed a hypoxia-related lncRNA prognosis prediction model, which can reliably and accurately predict the prognosis of gastric cancer (8).

Copper-induced cell death is different from other regulated cell death such as apoptosis, pyroptosis, and ferroptosis. Cells have a certain threshold for copper tolerance and excessive copper will lead to oxidative stress and cytotoxicity (9, 10). Accumulation and direct bindings of Cu in mitochondria drives the aggregation of lipoylated TCA cycle enzymes (11). In addition, cupper has been widely studied in drug development, which showed bright prospects in anticancer purposes (12). Therefore, further studies on the discovery of biomarkers from appropriate patient populations or in-depth knowledge of the molecule’s mechanisms are urgently needed (13).

There are numerous studies concentrating on structuring prediction models based on several types of programmed cell death. Interestingly, recent studies observed that the lncRNAs is correlated with programmed cell death in cancer cells (14, 15), suggesting a potential link between lncRNAs and cuproptosis. However, the role of cuproptosis-related lncRNAs in predicting prognosis in STAD has not been reported, and whether it can be used as an independent prognostic factor is not yet clear. Therefore, we analyze the data with a view to exploring cuproptosis-related biomarkers to predict the prognosis of STAD patients.

In this study, we combined the TCGA (The Cancer Genome Atlas) database to analyze the cuproptosis-related lncRNA data in STAD patients in order to explore new prognostic biomarkers of STAD patients. We constructed four prognosis-related signatures based on cuproptosis-related lncRNAs and assessed their ability to independently and accurately predict factors for STAD. Our study uncovers insights into underlying mechanisms of four cuproptosis-related lncRNAs and prognostic values in STAD. We also experimentally verified the modification relationship of two immune checkpoint genes by cuproptosis-related lncRNA.

## Materials and methods

### Data collection

We collected HTSeq-Count data, clinicopathological data, and OS information for gastric adenocarcinoma (STAD) from the TCGA database (16), the portal website is (https://portal.gdc.cancer.gov). In total, we downloaded transcriptomic data from 373 tissues (343 STAD tissues and 30 normal tissues) and collected clinicopathological features of 406 patients. Twenty cuproptosis-related genes were collected from the former literature (9, 10; **Table S1**). Additionally, we downloaded the annotated gene sets for Gene Set Enrichment Analysis (GSEA) from MSigDB4 (17).

### Screening of cuproptosis-related lncRNA

Based on R (ver. 4.2.0), we used the “limma” package for lncRNA and gene correlation analysis with a threshold (cor>0.4 and *p<* 0.001), and selected 432 lncRNA associated with cuproptosis by Pearson correlation analysis. We visualize the lncRNA-mRNA co-expression network associated with cuproptosis using the “ggalluvial” package.

### Construction of a prognostic model based on cuproptosis-related lncRNA

Based on R (ver. 4.2.0), the equivalent sampling method was adopted to divide the samples into training and test groups, the method was implemented by the “createDataPartition” function in the “caret” package, the potential value was set to 0.5, one sampling was made to obtain the random sampling data with a 1/2 ratio to the original dataset. Then, we used the “survival” and “glmnet” packages by minimum absolute shrinkage and selection operator (lasso) algorithm to analyze the 432 cuproptosis-related lncRNA to construct a prognostic model for STAD. Then, by using the prediction model, we calculated the risk score for each patient sample. The calculation formula is as follows:


$$Risk\;score=\sum_{i=1}^{n}Coefi*Xi$$

Where coefi is the coefficient, and xi is the expression of lncRNA.

The prognostic assessment model for OS was constructed according to risk scores.

### Risk model evaluation

Patients were divided into two groups: high risk and low risk groups, based on the median value of the risk factors. Their overall survival time differences were then compared using the Kaplan–Meier method, and receiver operating characteristics (ROC) curves were used to assess the specificity and sensitivity of the risk model. ROC curves of 1, 3, and 5 years were made in each group.

### Independent prognostic analysis of the risk models

Combining clinical characteristics with the risk assessment model of lncRNA for both univariate and multivariate COX regression analysis, the result *p<* 0.05 in the multivariate Cox analysis can be considered as an independent prognostic factor for STAD outcome.

### Functional enrichment analysis

A mRNA-lncRNA co-expression network was made to identify genes that correlate with necroptosis-related lncRNAs. We picked the top 174 genes with a threshold (cor>0.5 and *p<* 0.001) for functional analysis. The target genes were submitted to the website DAVID (https://david.ncifcrf.gov) to perform Gene Ontology (GO) annotation and Kyoto Encyclopedia of Genes and Genomes (KEGG) pathway enrichment analysis.

### Predictive ICB response and immune evasion of identified STAD subtypes by TIDE algorithm

Tumor Immune Dysfunction and Exclusion (TIDE) algorithm is a computational method using gene expression profile to evaluate tumor microenvironment (18). TIDE uses a set of gene expression markers to evaluate two essential mechanisms of tumor immune evasion, including dysfunction of tumor infiltrating cytotoxic T lymphocytes (CTL) and exclusion of CTL by immunosuppressive factors (18). Several studies have testified its utility in predicting the immune checkpoint blockade (ICB) therapy efficiency (19–22).

Based on TIDE algorithm, potential ICB response was predicted in both low risk and high risk groups.

### Immune checkpoint expression analysis associated with risk score

Based on studies by Hu et al. (23), co-expression network of the screened four lncRNAs and the collected immune checkpoint genes was established (*p<* 0.001). Then, by Wilcoxon test analysis (*p<* 0.01), differentially-expressed immune checkpoint genes between STAD risk types were screened out.

### Cell lines and cell culture

The human gastric cancer cell line SGC-7901 and AGS were purchased from American Type Culture Collection (ATCC). Both GC cell lines were identified by short tandem repeat analysis, and the results of mycoplasma test were negative and both cell lines were cultured with RPMI-1640 medium (Gibco, San Francisco, CA, USA) containing 10% fetal bovine serum (Gibco) with 100 U/ml penicillin and streptomycin (Gibco, Shanghai, China) at 37°C in a humidified incubator of 5% CO2.

### siRNA construction and infection

siRNA sequences targeting LINC01150 (siRNA, siLINC01150#1, and siLINC01150#2) was designed and constructed by GenePharma Co. Ltd., Shanghai, China. siRNA was added to the culture medium of ESCC cells by using riboFECT Transfection Kit (Ribobio, Shanghai, China). The targeted LINC01150 sequences were as follows: GGGAUAGGUAUUGUGGAAUTT (siLINC01150#1) and CUCCAUGCUUGCCUUAUCUTT (siLINC01150#2).

### RNA extraction and real−time qPCR

Total RNA was extracted from samples with RNAi Plus reagent (TAKARA, Japan) and quantified by using Nanodrop 8000 and stored at −80°C. 1000 ng of RNA was reversely transcribed into cDNA using a reverse transcription system (TAKARA, Japan). Real-Time qPCR was performed to quantify the transcripts using TB Green PCR Master Mix (TAKARA, Japan). The amplification primers for the LINC01150 coding region were as follows: GCGGGATAGGTATTGTGGAATGAGG (forward), TCTGAGACCGTGACTCCTGACTTC (reverse). The relative abundance of RNA was normalized to β-Actin. 2−(ΔΔCt) was used to calculate the relative abundance of mRNA.

### Western blotting

Cells were lysed in cold RIPA buffer containing protease inhibitors. Equal amounts of total protein were separated by SDS-PAGE transferred to PVDF membranes, and blocked in 5% milk. The primary antibodies were diluted according to the instructions. Membranes were incubated overnight with primary antibodies at 4°C, followed by secondary antibodies for 1 h at room temperature. Blots were developed using Millipore Immobilon Western Chemiluminescent HRP Substrate.

### Immunohistochemistry assay

Immunohistochemistry analysis was conducted, all paraffin-embedded material was sectioned at 4 μm. After dewaxing and hydration, the sections were incubated overnight with antibodies against human CD209 (A1466, Abclonal), human HAVCR2 (DF6979, Affinity). Primary antibody was detected with horseradish peroxidase-conjugated secondary antibodies incubated for 8 min. Sections were washed in distilled water, counterstained with hematoxylin, dehydrated, and mounted. The whole tissue section was scored with staining intensity and percentage and the scoring scale was graded as follows: 0 points (no staining), 1 point (light brown staining), 2 points (brown staining), and 3 points (dark brown staining). The percentage of positive cells is divided into four levels: 1 point (<5%), 2 points (5%–30%), 3 points (31%–60%), 4 points (61%–100%). The IHC staining score was calculated as follows: intensity score×percentage score.

### Statistical treatment

All statistical analyses of this study were performed in R language (ver. 4.2.0) with *p<* 0.05 as statistically significant.

## Results

### Clinical characteristics of the STAD patients

Clinical data on the detailed clinicopathological characteristics of the 406 STAD patients obtained from the TCGA database are shown in **Table 1**.

**Table 1:** Clinicopathological characteristics of 406 STAD patients obtained from the TCGA database.

Clinical characteristic	N	Percentage (%)
Age		
>60 years	274	67.49
≤60 years	129	31.77
Gender		
Male	256	63.05
Female	150	36.95
Stage		
I	56	13.79
II	118	29.06
III	167	41.13
IV	39	9.61
Tumor		
T1	23	5.67
T2	85	20.94
T3	185	45.57
T4	103	25.37
Lymph nodes metastasis		
N0	122	30.05
N1	109	26.85
N2	80	19.70
N3	78	19.21
Grade		
G1	10	2.46
G2	149	37.70
G3	240	58.97
Distant metastasis		
M0	361	88.91
M1	27	14.71

### Cuproptosis-related lncRNA-mRNA co-expression network

The expression of 20 cuproptosis-related genes in TCGA database was compiled according to previous studies, Pearson correlation analysis between cuproptosis-related genes and lncRNA in TCGA database was conducted, and cuproptosis-related lncRNAs were screened out (cor > 0.4, *p<* 0.001) to draw the Sanki diagram of lncRNA-mRNA co-expression network (**Supplementary Figure 1**).

### Construction of a lncRNA model for cuproptosis-related prognosis prediction

**Figure 1:**
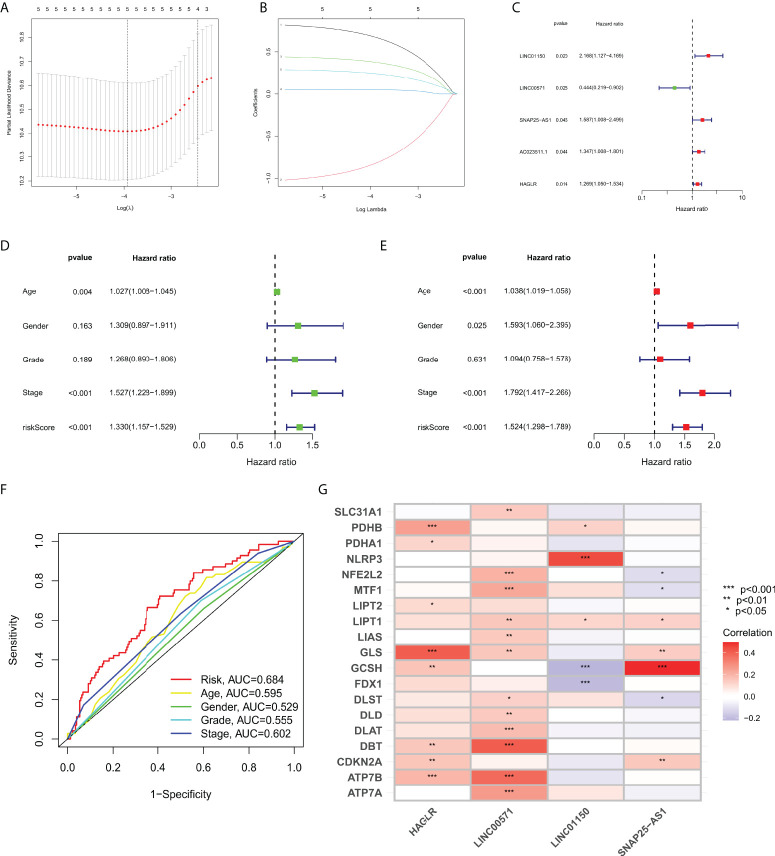
**(A)** LASSO Cox regression analysis revealed five cuproptosis-related lncRNAs based LASSO cross validation plot. **(B)** LASSO coefficient of eight cuproptosis-related lncRNAs in STAD. **(C)** Univariate Cox regression showed five cuproptosis-related lncRNAs. **(D)** Univariate Cox regression indicated that the age, and risk score were associated with OS (*p<* 0.05). **(E)** Multivariate Cox regression demonstrated that the age, N-stage, and risk score (*p<* 0.001) were independent prognostic indicators of OS in patients with STAD. **(F)** ROC curve showed that the risk score had the highest prognostic accuracy. **(G)** Correlation between cuproptosis-related genes and four lncRNAs constructed the nomogram. *p value<0.05, **p value<0.01, ***p value<0.001.

Samples were first randomly divided into train and test groups. Then, a prognostic model for gastric cancer patients based on four cuproptosis-related lncRNAs was constructed by univariate Cox regression analysis with the LASSO algorithm and multivariate Cox regression (**Figures 1A–C**). The Multivariate Cox result for cuproptosis-related lncRNAs based on TCGA-STAD is shown in **Table S2**.

### Correlation between the cuproptosis-related lncRNA prognosis model and the clinicopathological features

We performed Cox regression analysis to testify whether the prognostic model of cuproptosis-related lncRNAs is an independent risk factor for gastric cancer prognosis,. The results of univariate COX regression in **Figure 1D** showed that the model (*p<* 0.001), tumor grade (*p<* 0.001), pathological stage (*p =* 0.189), age (*p =* 0.004), and gender (*p =* 0.163) were important for prognostic prediction. Meanwhile, the multivariate Cox regression analysis showed that the model (*p<* 0.001, hazard ratio HR=1.524, 95%CI=1.298-1.789) would be an independent risk factor for gastric cancer prognosis (**Figure 1E**). AUC value of the ROC curve of all samples is 0.684, which is superior to traditional clinicopathological features in predicting STAD prognosis (**Figure 1F**). Correlation of cuproptosis-related genes were analyzed with four lncRNA that construct the nomogram (**Figure 1G**).

### Prognostic evaluation of cuproptosis-related lncRNA nomogram

**Figure 2:**
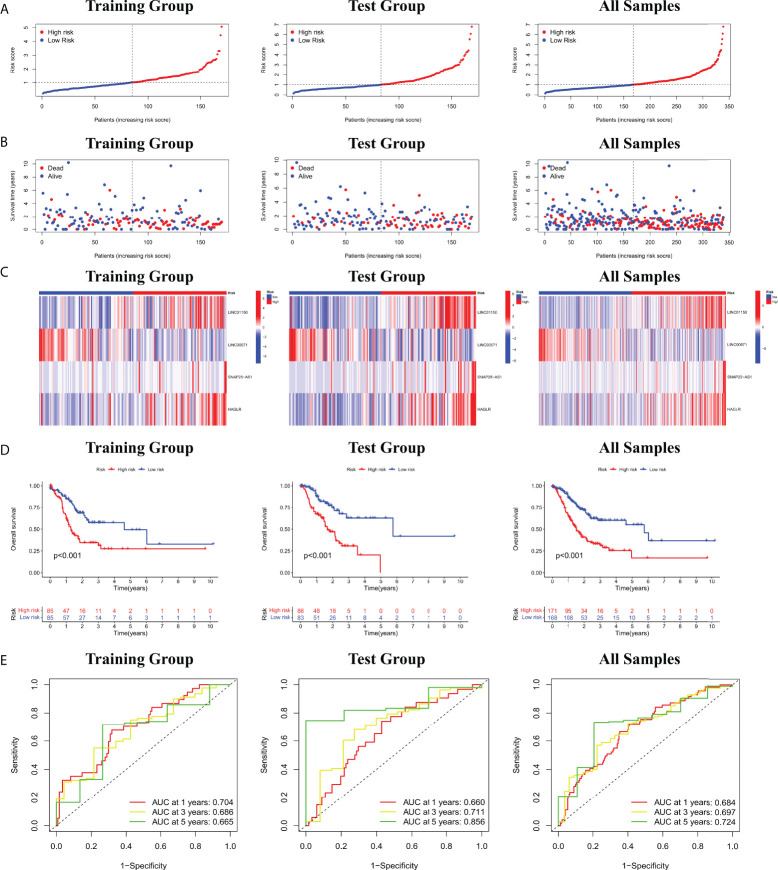
**(A)** Risk score distribution of patients with STAD based on cuproptosis-related lncRNAs. **(B)** Scatter plots showed the association between the OS and the risk score distribution. **(C)** Heat maps of expression of the four lncRNAs signals associated with cuproptosis. **(D)** K-M survival curve on OS status in STAD patients. **(E)** ROC curve of 1, 3, and 5 years in training and test groups (including all samples).

Based on four cuproptosis-related lncRNA models obtained in the previous period, we calculated the risk score for each patient sample. Patients were then divided into high risk (Risk score higher than median Risk score) and low risk (Risk score not higher than median Risk score) (**Figure 2A**). As shown in **Figure 2B**, more deaths in the high-risk group were observed from the scatter plots of the Risk score distribution (**Figure 2B**). The heat map of expression of the lncRNA signals associated with cuproptosis is shown in **Figure 2C**. Meanwhile, K-M survival curve analysis was used to evaluate the prognostic efficacy of this model on OS status in STAD patients. We found that OS was both in the train and test groups (including all samples) (*p<* 0.001; **Figure 2D**). We also evaluated the prognostic accuracy of this model using the ROC curve analysis. The results show that the AUC value of the ROC curve of all samples is 0.684, 0.697, 0.724 in 1, 3, and 5 years, respectively (**Figure 2E**). All these results collectively suggest that these four lncRNA signals associated with cuproptosis would be a valuable prognostic model for gastric cancer.

**Figure 3:**
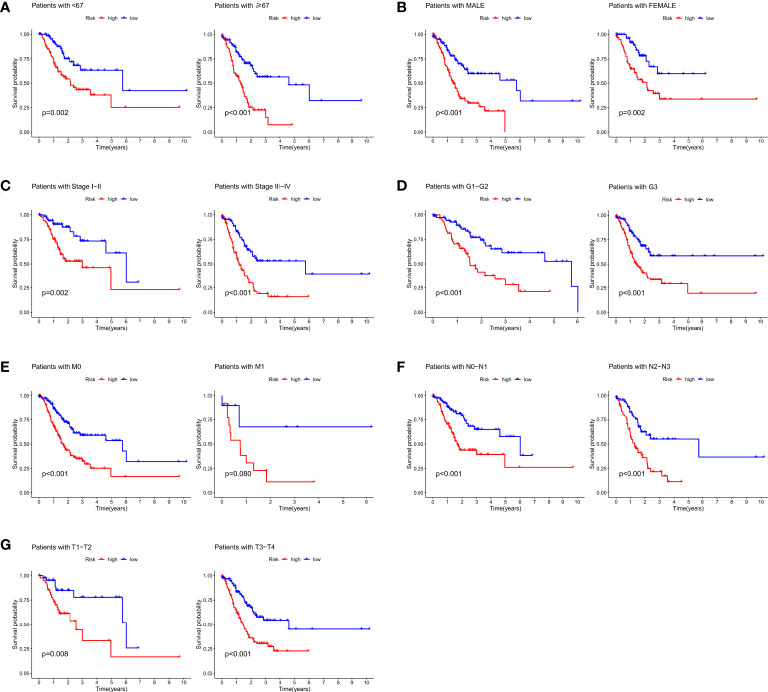
K-M survival analysis for clinicopathological subgroups divided by age **(A)**, sex **(B)**, clinical stage **(C)**, histological grade **(D)**, M **(E)**, N **(F)**, and T **(G)**.

Furthermore, the correlation between prognostic models based on four cuproptosis-related lncRNA and clinicopathological features was shown in **Figure S3**. We also performed a K-M survival analysis for each subgroup isolated according to the clinicopathological characteristics (**Figure 3**). Subgroups were divided by age, sex, T, N, M, clinical, and grade (as shown in **Figures 3A–G**, respectively). The OS rate was significantly lower in the high-risk population groups related to age, sex, T, N, M, clinical, and grade.

### Evaluation of the clinical utility of cuproptosis-related lncRNA nomogram

**Figure 4:**
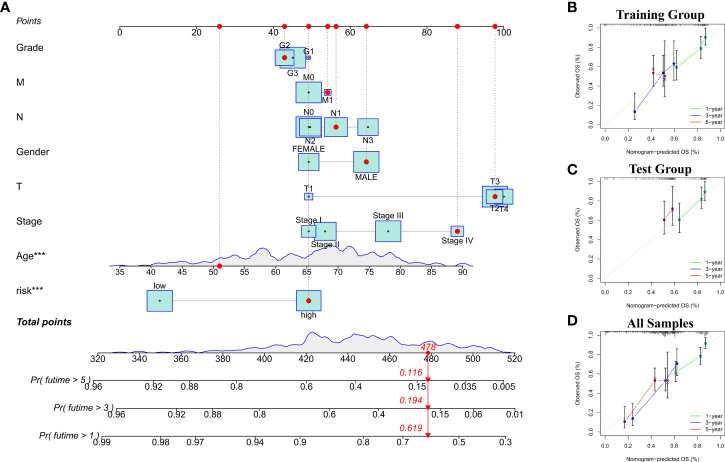
**(A)** Nomogram with Risk scores and clinicopathological features for risk score calculation. **(B–D)** The calibration curves showed the comparison between predicted and actual 1-, 3-, and 5-year OS in training and test groups (including all samples).

To evaluate the potential clinical utility of a prognostic model based on cuproptosis-related lncRNAs, we developed a nomogram with Risk scores and clinicopathological features to predict OS rates at 1, 3, and 5 years in gastric cancer patients. As shown in **Figure 4A**, the predicted prognosis was correlated with the calculated Risk score, where patients with higher calculated Risk score had worse predicted prognosis. We then constructed calibration curves to assess the agreement between the OS rate predicted by the nomograms and the actual observed OS rate. The results showed a relatively good fit to the 1-, 3-, and 5-year OS predictions in training and test groups (including all samples; **Figures 4B–D**).

### Functional annotation for the cuproptosis-related lncRNA nomogram

**Figure 5:**
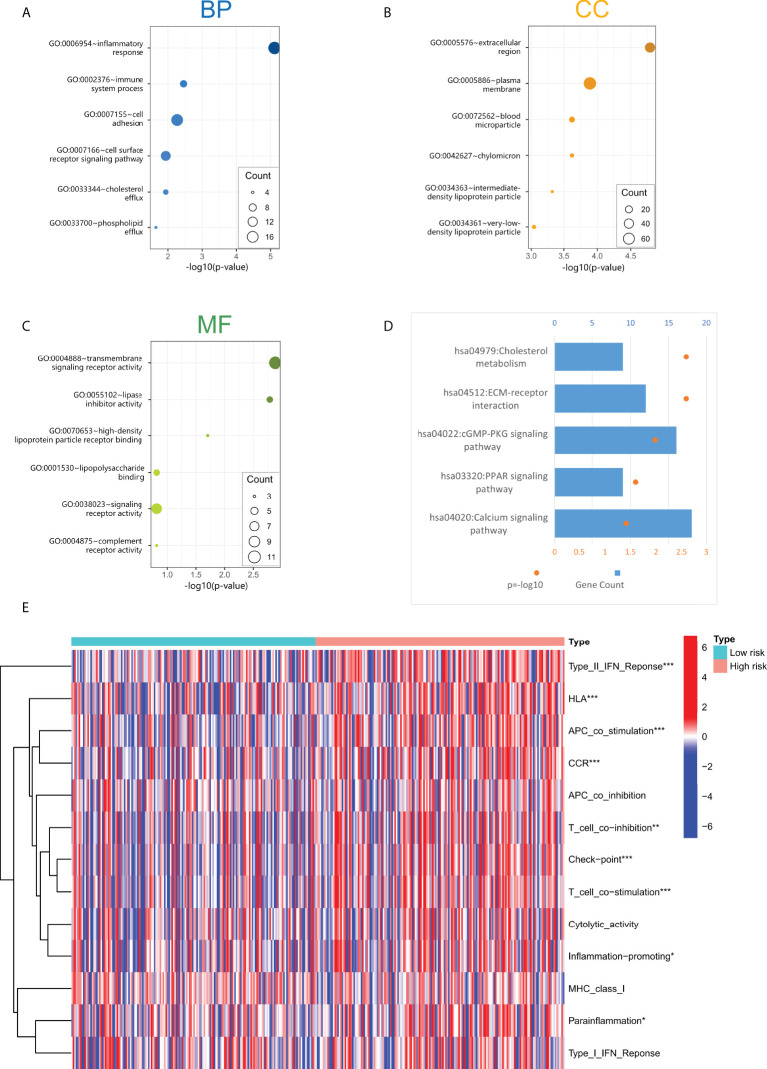
**(A–C)** The screened four lncRNAs related biological process (BP), cellular components (CC), and molecular function (MF). **(D)** KEGG pathway enrichment analysis of four cuproptosis-related lncRNAs. **(E)** Immune-related function analysis in high-risk and low-risk groups. "*" represents p value<0.05, "**" represents p value<0.01, "***" represents p<0.001.

We constructed a lncRNA-mRNA co-expression network based on Pearson correlation analysis to evaluate the biological function of cuproptosis-related lncRNA in this prediction model (**Table S4**). The significantly correlated genes (cor> 0.5, *p<* 0.001) were enriched for GO and KEGG analysis to elucidate their biological functions. The GO analysis includes the biological process (BP), cellular components (CC), and molecular function (MF) categories (**Figures 5A–C**). BP categories included inflammatory and immune responses, CC categories were mainly enriched in extracellular regions and cytoplasmic membranes, MF categories were mainly enriched in transcellular receptor signaling. KEGG pathway enrichment analysis (**Figure 5D**) indicated that these genes are enriched with tumor-related signaling pathways, including cholesterol metabolism, ECM-receptor interaction, cGMP-PKG signaling, and PPAR signaling pathway, suggesting that the activation of these pathways may increase the risk of death in patients.

### Analysis of the immune-related functions of the cuproptosis-related lncRNA model

Through GO and KEGG enrichment analyses, it was found that the functions of lncRNAs in the cuproptosis-associated risk model may be related to the immune response process. In addition, we performed immune-related function analysis in high-risk and low-risk groups to screen out significant differences (**Figure 5E**). The results showed that the main differential immune-related functions were TypeII-IFN Response, HLA, APC co-stimulation, CCR, and T cell co inhibition. These indicated functions may increase the risk of death in GC patients.

### Identification of ICB response and immune evasion of STAD subtypes by TIDE

**Figure 6:**
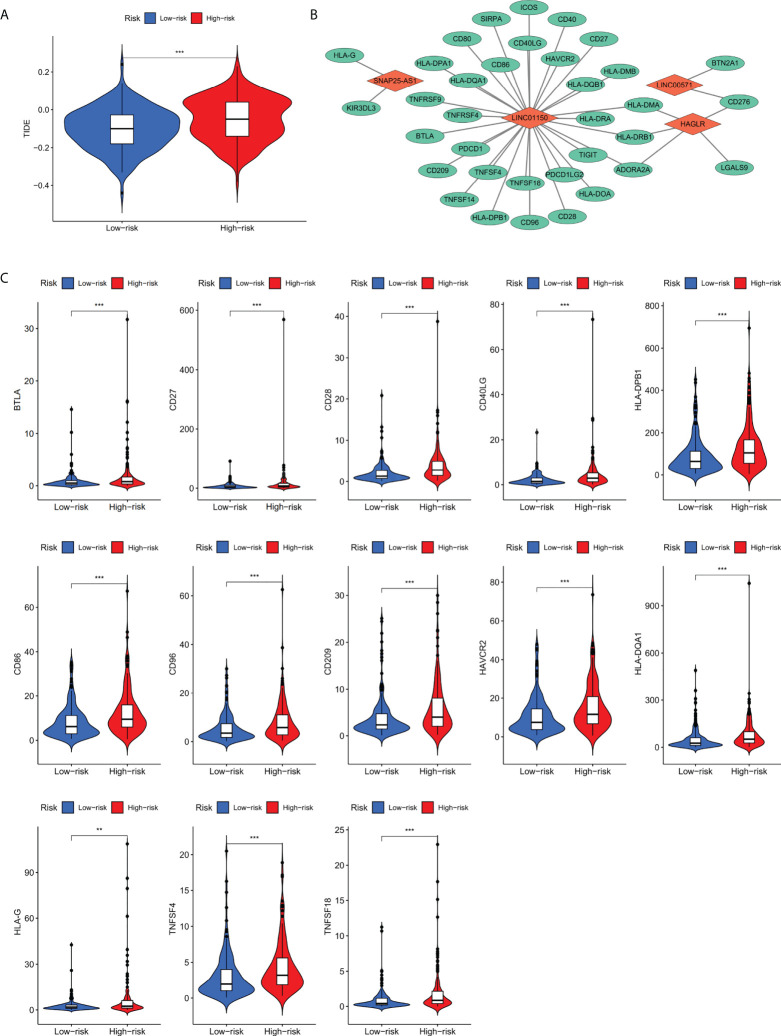
**(A)** TIDE scoring for immune evasion and ICB resistance in high-risk and low-risk groups. **(B)** The co-expression network between four screened lncRNAs with immune checkpoint genes. **(C)** Thirteen differentially expressed immune checkpoint genes co-expressed with four cuproptosis-related lncRNAs. **p value<0.01,***p value<0.001.

We scored TIDE for immune evasion and immunotherapy tolerance based on gene expression in patients with high-risk and low-risk groups, and the results showed that TIDE scores were significantly higher in high-risk group than that in low-risk group (*p<* 0.001; **Figure 6A**), suggesting that patients in high-risk group exert higher tolerance of ICB and higher possibility of immune evasion. Therefore, patients in low risk group are supposed to be more effective for ICB treatment.

### Analysis of the risks core-associated immune checkpoint genes

Based on TIDE scoring system, it was concluded that patients in the high risk group had the potential of ICB tolerance and immune evasion. Subsequently, co-expression network of the screened four lncRNAs and the collected immune checkpoint genes was established. We found that out of 79 immune checkpoint-related genes, 35 genes were correlated the four screened lncRNAs (*p<* 0.001). The network between four lncRNAs with immune checkpoint genes is shown in **Figure 6B**.

To further analyze immune checkpoint genes that associate with risk score, the expression profiles of 35 genes were analyzed between high risk and low risk groups, it was found that BTLA, CD27, CD28, CD40LG, CD86, CD96, CD209, HAVCR2, HLA-DPB1, HLA-DQA1, HLA-G, TNFSF4, and TNFSF18 (*p<* 0.01; **Figure 6C**) were differentially expressed. By combining the results above, we speculate that these 13 immune checkpoint genes may be regulated by the four cuproptosis-related prognostic lncRNAs and is also relevant to the prognosis of gastric cancer patients.

### LINC01150 regulates the expression of immune checkpoint genes CD209 and HAVCR2

**Figure 7:**
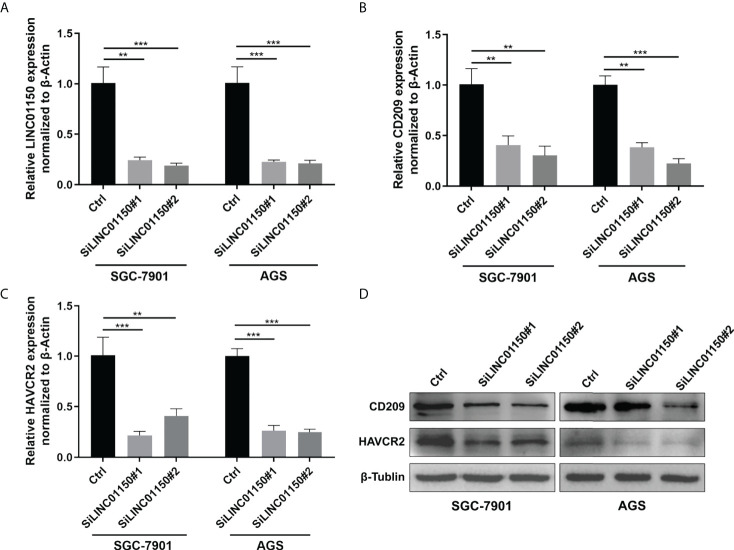
**(A–C)** RT-qPCR analysis of LINC01150, CD209, HAVCR2 expressions in GC cells after siRNA knock down of LINC01150. **(D)** Western Blot analysis of CD209, HAVCR2 expressions in GC cells after siRNA knock down of LINC01150.

Based on our previous findings of cuproptosis-related lncRNA and its correlation with immune checkpoint genes, two genes with the minimum p value: HAVCR2 and CD209 was selected to testify the relationship with LINC01150. We examined the effect of silencing LINC01150 expression (**Figure 7A**) in gastric cancer cell lines SGC-7901 and AGS. RT-qPCR (**Figures 7B, C**) and Western Blotting (**Figure 7D**) results demonstrated that depletion of LINC01150 consistently resulted in reduction of CD209 and HAVCR2 both in mRNA and protein levels in GC cells.

### CD209 and HAVCR2 were overexpressed in human GC and predicts poor survival

**Figure 8:**
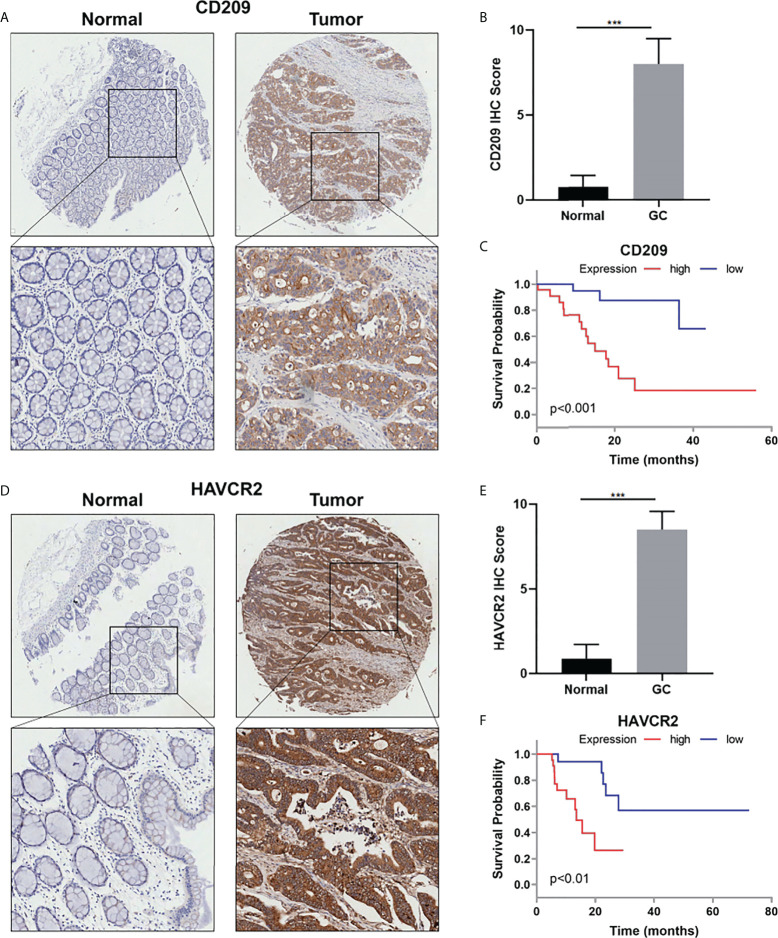
**(A)** Representative IHC staining for CD209 in GC and adjacent non-tumor tissues. **(B)** Quantification of **(A)**. **(C)** The overall survival analysis was plotted using Kaplan–Meier Plotter for patients with GC by IHC level of CD209. **(D)** Representative IHC staining for HAVCR2 in GC and adjacent non-tumor tissues. **(E)** Quantification of **(D)**. **(F)** The overall survival analysis was plotted using Kaplan–Meier Plotter for patients with GC by IHC level of HAVCR2. ***p value<0.001.

IHC staining was performed to examine CD209 and HAVCR2 protein levels in GC tissues and corresponding non-tumor tissues. The IHC results showed that the average expression score of CD209 and HAVCR2 was significantly higher in epithelial tissues of GC than in adjacent non-tumor counterparts (**Figures 8A, B, D, E**). In addition, Kaplan–Meier Plotter analysis showed that CD209 and HAVCR2 expressions were negatively correlated with the survival of GC patients (**Figures 8C, F**) .

## Discussion

Gastric adenocarcimoma (STAD) is the most common histological type of GC (24) and there is a lack of recognized and effective indicator to judge the prognosis of STAD patients (25). LncRNAs are effective indicators of tumor prognosis. Luo et al. constructed a necroptosis-related prognostic signature comprising 12 lncRNAs to predict OS and DFS in stomach adenocarcinoma (14). Pan et al. found 17 ferroptosis-related lncRNA signature in gastric cancer with prognostic value (15). Cuproptosis is a copper-dependent regulated cell death (RCD) mode, which mainly dependent on copper ion-dependent mitochondrial stress to regulate cell death, especially the aggregation of lipoylated mitochondrial enzymes and the loss of Fe-s cluster proteins (9, 10). Direct bindings of Cu in mitochondria in the aggregation of TCA cycle enzymes may play vital roles in tumorigenesis (13). Therefore, we suppose that cuproptosis-related lncRNAs have crucial impacts on oncogenesis and prognosis.

Through GO functional analysis, we found that functions such as inflammatory response, immune response, and transmembrane cell signaling were significantly enriched. Through KEGG pathway enrichment analysis, we found significant enrichment in cholesterol metabolism, ECM-receptor, cGMP-PKG, PPAR, and calcium signaling pathways. Increased cholesterol biosynthesis in the lipid-and/or oxygen-limited tumor microenvironments is the characteristic of numerous malignancies (26). Enhanced cholesterol metabolism contributes to cancer progression, including cell proliferation (27), apoptosis (28), migration, and invasion (29). Additionally, increased efflux of cholesterol can shape the immunosuppressive tumor microenvironment and promote tumor-associated macrophage-mediated tumor progression (30). Alternatively, cholesterol in the tumor microenvironment induces CD8+ T cell expression of immune checkpoints and accelerates CD8+T cell depletion (31). Thus, cholesterol metabolism pathways may play important roles in various cellular and physiological activities, such as tumor microenvironment, immune evasion, cell proliferation, migration, and invasion, which are crucial characteristics for tumor progression. The ECM signaling pathway was identified as an possible pathway for EMT-related gene signature in previous studies of gastric cancer, and was used as a OS analysis marker in gastric cancer patients (32). Nucleotide *de novo* synthesis can increase the stemness and metastasis of breast cancer through cGMP-PKG-MAPK signaling pathway (33), and DARS-AS1/ATP1B2 regulates the progression of cervical cancer by modulating the cGMP-PKG pathway (34). PPARs act as fat sensors to regulate the transcription of lipid metabolic enzymes, and are closely related to energy (lipid and sugar) metabolism, cell differentiation, proliferation, apoptosis, and inflammatory reactions (35). PPARγ agonists showed dose-dependent inhibitory effects on the proliferation of the gastric cancer cells (36). In short, these pathways that we have enriched may be related to functions of cuproptosis, and our findings may provide new ideas for further research in cuproptosis in the future.

Since our findings in GO and KEGG analysis are mostly related to immune process, we hypothesized that our risk model is correlated mainly with immune functions. As a result, we analyzed the immune-related functional differences in the risk group and found significant differences in TypeII-IFN Response, HLA, APC co-stimulation, CCR, and T cell co-inhibition. The most prominent differential function was the TypeII-IFN Response. In previous studies, chronic activation of the TypeII-IFN pathway in previous studies suggests an association with immune evasion in prostate cancer (37). Activation of the TypeII-IFN signaling pathway can inhibit the transport of circulating T cells to the tumor by inhibiting calcium influx and inhibiting the activation of the kinases ERK and AKT (38). Moreover, after analyzing each group with TIDE scoring system, results showed that the high-risk group was featured by higher TIDE score, which on behalf of higher possibility of immune evasion and immunotherapy resistance (18).

Immune escape is the main cause of failure in tumor immunotherapy and immune checkpoints play essential roles in immune functions. In this study, 13 differentially expressed immune checkpoint genes for cuproptosis-related lncRNAs between two risk groups were screened out through immune checkpoint expression analysis. Our study testified that cuproptosis-related LINC01150 regulates the expression of immune checkpoint genes CD209 and HAVCR2. CD209 has been reported to be correlated with M2 like macrophage infiltration in GC (39). HAVCR2 was reported to mediate T-cell depletion and immune dysfunction (40). Both immune checkpoint genes are overexpressed in GC tissues and predicts poor survival in GC patients. These findings may provide new insights into immunotherapy strategies to improve STAD patients’ prognosis, and indicates underlying molecular mechanisms between cuproptosis-related lncRNAs and immunogenic molecules.

In our risk score model, the ROC values for 1, 3, and 5 years are 0.684, 0.697, and 0.724, respectively, which indicates that the nomogram has relatively higher accuracy in predicting long-term outcomes. In addition, patients in the high-risk group had the higher potential of ICB tolerance and immune evasion.

However, this paper also has some limitations. The data we used to construct the prognostic evaluation model are from the TCGA database with a single data source. In the future, we will include more clinical data to verify the generality and accuracy of the research results.

## Conclusion

In this study, we, for the first time, correlated cuproptosis- related lncRNA with the prognosis of STAD patients to construct a prognostic evaluation model based on TCGA database. The prognostic model can not only predict patient survival and prognosis, but can also predict ICB sensitivity and tumor immunological features.

## Data availability statement

The original contributions presented in the study are included in the article/**Supplementary Material**. Further inquiries can be directed to the corresponding authors.

## Author contributions

MX and TC conceived and designed the study. AF conducted the bioinformatic analysis. AF and LH wrote the original draft of the manuscript. MX and TC contributed to draft revising and fund providing. LH participated in methodology supervision and draft revising. All authors contributed to the article and approved the submitted version.

## Funding

This work was supported by Medical discipline Construction Project of Pudong Health Committee of Shanghai (PWYgf2021-02).

## Conflict of interest

The authors declare that the research was conducted in the absence of any commercial or financial relationships that could be construed as a potential conflict of interest.

## Publisher’s note

All claims expressed in this article are solely those of the authors and do not necessarily represent those of their affiliated organizations, or those of the publisher, the editors and the reviewers. Any product that may be evaluated in this article, or claim that may be made by its manufacturer, is not guaranteed or endorsed by the publisher.

## Supplementary material

Supplementary Figure 1 Sanki diagram of lncRNA-mRNA co-expression network.
