# Supplementary material for: A novel cuproptosis-related lncRNA nomogram to improve the prognosis prediction of gastric cancer
Source: Front Oncol. 2022 Aug 29;12:957966. doi: 10.3389/fonc.2022.957966 (PMC9465020; doi:10.3389/fonc.2022.957966)
Supplement: Supplementary file 1 [file Table_1.docx]

**Supplement Table 1** Human cuproptosis-related genes

| **Entrez ID** | **Gene Symbol** | **Gene name** |
| --- | --- | --- |
| 4780 | NFE2L2 | NFE2 like bZIP transcription factor 2 |
| 114548 | NLRP3 | NLR family pyrin domain containing 3 |
| 540 | ATP7B | ATPase copper transporting beta |
| 538 | ATP7A | ATPase copper transporting alpha |
| 1317 | SLC31A1 | solute carrier family 31 member 1 |
| 2230 | FDX1 | ferredoxin 1 |
| 11019 | LIAS | lipoic acid synthetase |
| 51601 | LIPT1 | lipoyltransferase 1 |
| 387787 | LIPT2 | lipoyl(octanoyl) transferase 2 |
| 1738 | DLD | dihydrolipoamide dehydrogenase |
| 1737 | DLAT | dihydrolipoamide S-acetyltransferase |
| 1743 | DLST | dihydrolipoamide S-succinyltransferase |
| 5160 | PDHA1 | pyruvate dehydrogenase E1 subunit alpha 1 |
| 5162 | PDHB | pyruvate dehydrogenase E1 subunit beta |
| 4520 | MTF1 | metal regulatory transcription factor 1 |
| 2744 | GLS | glutaminase |
| 1029 | CDKN2A | cyclin dependent kinase inhibitor 2A |
| 1629 | DBT | dihydrolipoamide branched chain transacylase E2 |
| 2653 | GCSH | glycine cleavage system protein H |
| 1743 | DLST | dihydrolipoamide S-succinyltransferase |
