# Supplementary material for: A novel cuproptosis-related lncRNA nomogram to improve the prognosis prediction of gastric cancer
Source: Front Oncol. 2022 Aug 29;12:957966. doi: 10.3389/fonc.2022.957966 (PMC9465020; doi:10.3389/fonc.2022.957966)
Supplement: Supplementary file 2 [file Table_2.docx]

Table S2

| ID | Coef |
| --- | --- |
| LINC01150 | 0.848321781451102 |
| LINC00571 | -1.07315703689897 |
| SNAP25-AS1 | 0.513511858686925 |
| HAGLR | 0.299416667417174 |

**Table S2:** Multivariate Cox results for cuproptosis-related lncRNAs based on TCGA-STAD.
