# Supplementary figures and images for: A novel cuproptosis-related lncRNA nomogram to improve the prognosis prediction of gastric cancer
Source: Front Oncol. 2022 Aug 29;12:957966. doi: 10.3389/fonc.2022.957966 (PMC9465020; doi:10.3389/fonc.2022.957966)

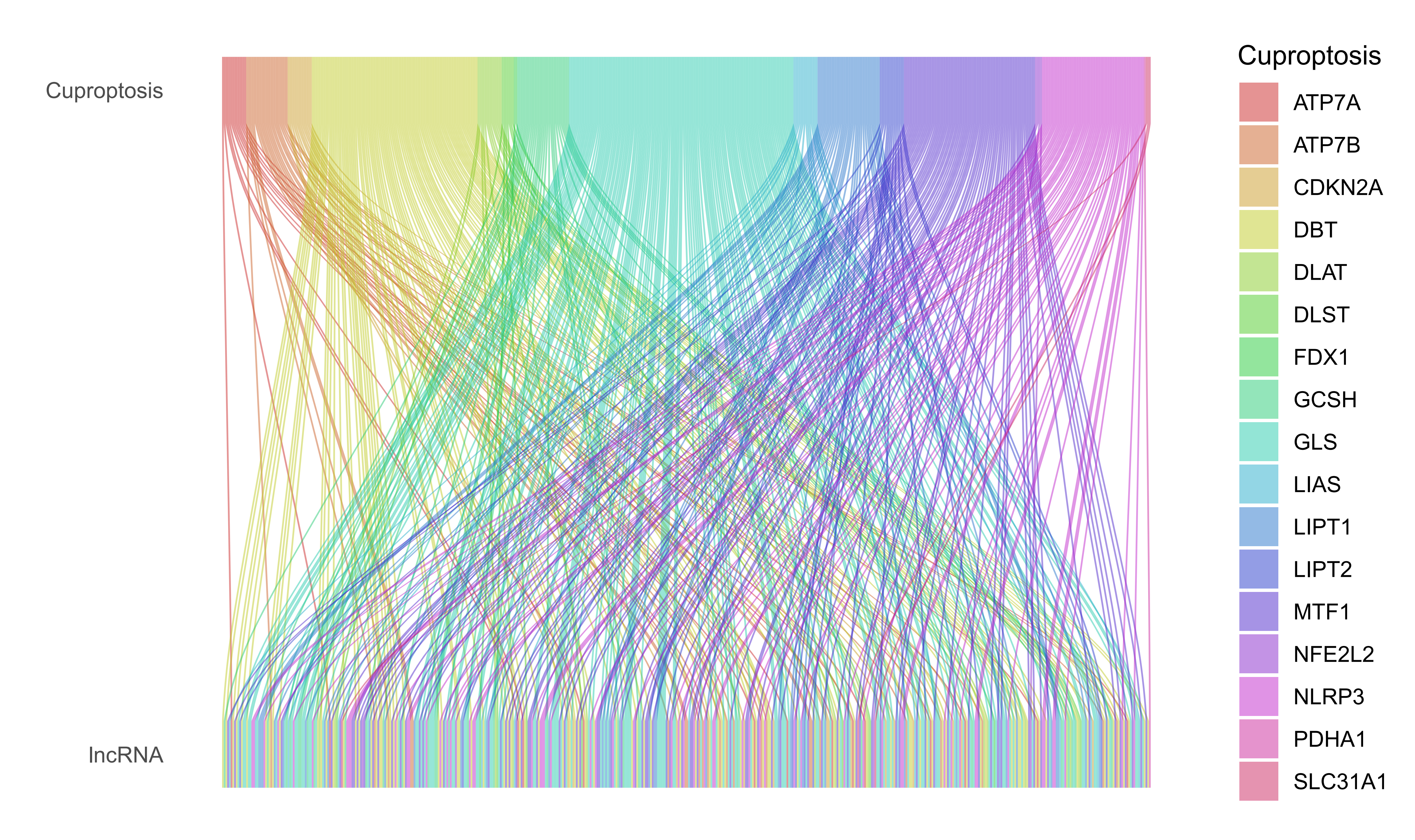

Supplement: Supplementary Figure 1 — Sanki diagram of lncRNA-mRNA co-expression network. [file Image_1.tif]
